# Seroprevalence of Heartland Virus Antibodies in Blood Donors, Northwestern Missouri, USA

**DOI:** 10.3201/eid2502.181288

**Published:** 2019-02

**Authors:** Nicole P. Lindsey, Jay E. Menitove, Brad J. Biggerstaff, George Turabelidze, Pat Parton, Kim Peck, Alison J. Basile, Olga I. Kosoy, Marc Fischer, J. Erin Staples

**Affiliations:** Centers for Disease Control and Prevention, Fort Collins, Colorado, USA (N.P. Lindsey, B.J. Biggerstaff, A.J. Basile, O.I. Kosoy, M. Fischer, J.E. Staples);; Community Blood Center of Greater Kansas City, Kansas City, Missouri, USA (J.E. Menitove, P. Parton, K. Peck);; Missouri Department of Health and Senior Services, St. Louis, Missouri, USA (G. Turabelidze)

**Keywords:** Heartland virus, viruses, arbovirus, phlebovirus, seroprevalence, antibodies, blood donors, Missouri, United States

## Abstract

We estimated the seroprevalence of Heartland virus antibodies to be 0.9% (95% CI 0.4%–4.2%) in a convenience sample of blood donors from northwestern Missouri, USA, where human cases and infected ticks have been identified. Although these findings suggest that some past human infections were undetected, the estimated prevalence is low.

In 2012, Heartland virus, a novel virus in the family *Phenuiviridae*, genus *Phlebovirus*, was identified in blood specimens obtained from 2 residents (men) of northwestern Missouri, USA (*1*). Given the clinical manifestations of illness and history of tick bites of the patients, both men were initially believed to have ehrlichiosis but they failed to improve after being given doxycycline.

Before identification of Heartland virus in these 2 patients, to our knowledge, there were no known phleboviruses that caused human disease in the United States (*1**,**2*). Subsequent field work identified *Amblyomma americanum* ticks, which are widely distributed across the eastern and central United States, as the likely vector for the virus (*3**,**4*). Wild animals in Florida, Georgia, Illinois, Indiana, Kansas, Kentucky, Maine, Missouri, New Hampshire, North Carolina, Tennessee, Texas, and Vermont have been found to be seropositive for Heartland virus antibodies (*5*). Investigations are underway to identify more disease cases, but little is known about the incidence of Heartland virus infection in humans. The objective of this study was to estimate the seroprevalence of antibodies against Heartland virus in a convenience sample of blood donors who reside in northwestern Missouri where human cases and infected ticks have been identified (*1**,**3**,**6*).

## The Study

Because the anticipated seroprevalence of Heartland virus was unknown, we calculated a sample size that would enable us to conclude with reasonable confidence that infections were rare in case no positive results were detected. To this end, we calculated that serum from 500 individual blood donors was required to infer that the true prevalence was <0.5% with 95% confidence. In addition, this sample size ensured that the prevalence would be estimated with precision no worse than ± 4.5% with 95% confidence.

Most blood donors in northwestern Missouri donate through community blood drives operated by the Community Blood Center of Greater Kansas City (Kansas City, MO, USA). Specimens were collected from consecutive blood drives conducted during November 4–December 3, 2013. The study population included blood donors >16 years of age who had adequate residual specimens remaining after standard screening was performed. We originally intended to include residents of 15 counties surrounding the area where the first cases were identified (Figure). However, because 5 of those counties had <5 donations, analysis was restricted to residents of the remaining 10 counties. At the time of donation, blood donors consented to have residual specimen used for research. If a donor did not provide this consent, their sample was excluded. All specimens were deidentified before shipment to the Centers for Disease Control and Prevention (CDC; Fort Collins, CO, USA) for testing. The only data included with the specimens were patient age, sex, and county of residence. Testing of deidentified, residual samples was deemed by CDC to not involve human subjects under 45 CFR 46.102 (https://www.hhs.gov/ohrp/regulations-and-policy/regulations/regulatory-text/index.html#46.102), and human subjects regulations were not applicable.

**Figure:**
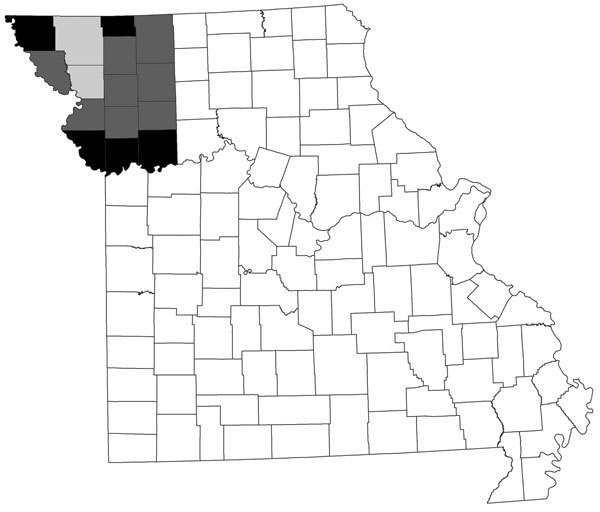
Location of counties targeted for study of seroprevalence of Heartland virus in blood donors, northwestern Missouri, USA. Gray shading indicates 10 counties included in analysis; lighter gray shading indicates counties where first cases were identified. Black shading indicates 5 counties excluded from analysis because they had <5 blood donors.

We screened all serum specimens for IgG against Heartland virus by using a Clinical Laboratory Improvement Amendments–approved microsphere assay as described (*7*). For specimens yielding IgG-positive results, we performed a more specific plaque reduction neutralization test (PRNT), which can differentiate between related phleboviruses in the United States, by using Vero E6 cells to confirm the presence of virus-specific neutralizing antibodies and a 90% plaque reduction criteria (*8**,**9*). We calculated seroprevalence by using the 2013 US Census midyear population estimate for persons >16 years of age for the area.

For the 487 blood donors tested, median age was 52 years (range 16–87 years), and 225 (46%) were men. Twelve serum specimens were positive for IgG against Heartland virus, and 7 of those were confirmed for Heartland virus neutralizing antibodies by PRNT. For the 7 donors with Heartland virus neutralizing antibodies, median age was 33 years (range 30–78 years) and 4 (57%) were men. Five (71%) of the 7 positive persons were residents of Daviess County.

Because there were differences in the rates of blood donors per population in the included counties, we computed the estimate of the seroprevalence within the region by using a stratum-weighted estimate and 95% CI (*10*). We estimated a seroprevalence of 0.9% (95% CI 0.4‒4.2%) in blood donors >16 years of age in the 10-county region. Assuming this seroprevalence estimate was representative of the general population in the study region, we estimate that 1,431 (95% CI 660–6,708) adult residents in the area had been previously exposed to Heartland virus.

The findings of this analysis are subject to several limitations. Blood donors differ from the general population in age (>16 years), sex, health status, and potentially exposures. Therefore, these results might not be applicable to the general population in northwestern Missouri. For instance, 46% of our donors were men, compared with 51% of persons >18 years of age who live in 10-county areas included in our analysis. Furthermore, because we excluded counties without an adequate number of donors, data collected might not be representative of the entire region of northwestern Missouri that included counties in or near where human disease cases and infected ticks have been identified.

Because blood donors are required to not have had a recent illness and no information was collected regarding previous illnesses, we did not test for evidence of acute infection and cannot state whether identified infections were asymptomatic or might have resulted in symptomatic disease. In addition, because we were only identifying evidence of past infections to determine the seroprevalence in the area, we do not know the timing of identified human infections and whether these persons were infected in their county of residence.

## Conclusions

We estimated a prevalence of 0.9% for Heartland virus infection in northwestern Missouri, where the virus is known to have circulated. These results suggest that several infections have gone unidentified because they were asymptomatic or the infected persons did not seek care, were not tested, or were ill before the identification of Heartland virus as a cause of human disease. The finding that most identified infections were in 1 county could be a chance occurrence but also might suggest that the virus is geographically focally distributed.

On the basis of available data for >30 reported cases of Heartland virus disease, healthcare providers should consider testing for patients who have an acute febrile illness and leukopenia or thrombocytopenia not explained by another condition or who were suspected to have another tickborne disease but did not improve after appropriate treatment (e.g., doxycycline) (*6**,**11*). Testing should be limited to patients who resided in or traveled to an area with previous evidence of Heartland virus or had a known tick exposure (*5**,**6*).

Because Heartland virus is transmitted by infected ticks, prevention depends on using insect repellents, wearing long sleeves and pants, avoiding bushy and wooded areas, and performing tick checks after spending time outdoors. The clinical spectrum of Heartland virus disease remains to be described, including determination of whether asymptomatic infections can occur. In addition, research is needed to determine whether there are other modes of transmission for Heartland virus, including bloodborne transmission.
